# Reflections, impact and recommendations of a co-produced ecological momentary assessment (EMA) study with young people who have experience of suicidality and psychiatric inpatient care

**DOI:** 10.1192/j.eurpsy.2022.649

**Published:** 2022-09-01

**Authors:** L. Dewa, S. Pappa, L. Mitchell, M. Hadley, J. Cooke, P. Aylin

**Affiliations:** 1 Imperial College London, School Of Public Health, London, United Kingdom; 2 West London NHS Trust, West London Nhs Trust, London, United Kingdom

**Keywords:** Ecological Momentary Assessment, Suicide, Transition, sleep

## Abstract

**Introduction:**

Patient and public involvement (PPI) in suicide research is ethical, moral and can deliver impact. However, inconsistent reporting of meaningful PPI, and hesitancy in sharing power with people with experience of suicidality (i.e.co-researchers) in research makes it difficult to understand the full potential impact of PPI on the research, researchers and co-researchers.

**Objectives:**

To describe how our ecological momentary assessment (EMA) study, examining the sleep-suicide relationship in young psychiatric inpatients (aged 18-35) transitioning to the community, has been co-produced, whilst reflecting on impact, challenges, and recommendations.

**Methods:**

We built on our experience of co-produced mental health research to conduct meaningful PPI in our study. Young adults with experience of psychiatric inpatient care and suicidality were appointed November 2020 to work across all research stages. Reflections on challenges, recommendations and impact have been collected throughout.

**Results:**

Three young people became co-researchers. Researcher and co-researcher reflections indicated establishing and maintaining safe environments for open discussion, and continued communication (e.g.WhatsApp group) were vital to effectively share power and decision making. Safeguarding and support requirements for both co-researchers (e.g.individualised strategy) and researcher (e.g.clinical supervision) were particularly evident. To date, the co-produced recruitment poster, research documentation, and research article have demonstrated significant impact.

**Conclusions:**

This is the first EMA study focused on suicide-sleep during transitions to be co-produced with young people with experience of suicidality. Co-producing suicide research is intensive, time-consuming, and challenging but makes a significant impact to the research, researchers, and co-researchers. We expect our learning will directly influence, and help others produce, meaningful co-produced suicide research.

**Disclosure:**

No significant relationships.

